# Quality Evaluation of *Mahonia bealei* (Fort.) Carr. Using Supercritical Fluid Chromatography with Chemical Pattern Recognition

**DOI:** 10.3390/molecules24203684

**Published:** 2019-10-13

**Authors:** Yang Huang, Zhengjin Jiang, Jue Wang, Guo Yin, Kun Jiang, Jiasheng Tu, Tiejie Wang

**Affiliations:** 1 Shenzhen Institute for Drug Control, Shenzhen 518057, China; huang_yang1987@hotmail.com (Y.H.);; 2 State Key Laboratory of Natural Medicines, Department of Pharmaceutics, China Pharmaceutical University, Nanjing 210009, China; 3 Institute of Pharmaceutical Analysis, College of Pharmacy, Jinan University, Guangzhou 510632, China; 4 Shenzhen Key Laboratory of Drug Quality Standard Research, Shenzhen 518057, China

**Keywords:** *M. bealei*, SFC fingerprint, chemical pattern recognition, quality evaluation

## Abstract

*Mahonia bealei* (Fort.) Carr. (*M. bealei*) plays an important role in the treatment of many diseases. In the present study, a comprehensive method combining supercritical fluid chromatography (SFC) fingerprints and chemical pattern recognition (CPR) for quality evaluation of *M. bealei* was developed. Similarity analysis, hierarchical cluster analysis (HCA), principal component analysis (PCA) were applied to classify and evaluate the samples of wild *M. bealei*, cultivated *M. bealei* and its substitutes according to the peak area of 11 components but an accurate classification could not be achieved. PLS-DA was then adopted to select the characteristic variables based on variable importance in projection (VIP) values that responsible for accurate classification. Six characteristics peaks with higher VIP values (≥1) were selected for building the CPR model. Based on the six variables, three types of samples were accurately classified into three related clusters. The model was further validated by a testing set samples and predication set samples. The results indicated the model was successfully established and predictive ability was also verified satisfactory. The established model demonstrated that the developed SFC coupled with PLS-DA method showed a great potential application for quality assessment of *M. bealei*.

## 1. Introduction

*Mahonia bealei* (Fort.) Carr. (*M. bealei*) is a well-known traditional Chinese medicine (TCM) and has been used for the treatment of acute dysentery, icteric hepatitis conjunctivitis, etc. [1]. Nowadays, over 50 species of *Mahonia* plant grow in China. Several *Mahonia* plants such as *Mahonia breviracema* Y. S. Wang et Hsiao (*M. breviracema* Y. S. Wang et Hsiao), *Mahonia duclouxiana* Gagnep (*M. duclouxiana* Gagnep), *Mahonia bodinieri* Gagnep (*M. bodinieri* Gagnep), and *Mahonia fordii* Schneid (*M. fordii* Schneid) were utilized as substitutes of *M. bealei* in Chinese folk medicine [2,3]. In addition, with increasing medicinal use demand, the wild *M. bealei* has been overexploited. Therefore, cultivated *M. bealei* has become the main source to satisfy the market demand. To our knowledge, the cultivation environment might affect the chemical content compared to the wild growing environment, resulting in quality differences [4]. As far as we know, little attention has been paid to the quality differences between wild and cultivated *M. bealei*. The current Chinese Pharmacopoeia describes several alkaloids, including columbamine, jatrorrhizine, palmatine, and berberine, as the main bioactive compounds in *M. bealei*. These compounds could be considered as chemical markers for the quality control of *M. bealei* [1]. However, it was revealed that many common components (i.e. berberine, jateorhizine, palmatine) [5] have been reported in *Mahonia* species. Owing to the complex composition of *Mahonia* species, it is insufficient to perform quality assessment using only several chemical markers. Therefore, a method for comprehensive and effective evaluation of *M. bealei* is necessary based on their integral components. 

Due to the typical complexity in composition of TCMs, fingerprint analysis have been accepted as an effective tool for evaluating the quality of TCMs without sacrificing reference standards that are usually highly expensive and far from need, such as high performance liquid chromatography (HPLC) [6,7], gas chromatography (GC) [8], near infrared spectroscopy (NIR) [9], high performance thin layer chromatography (HPTLC) [10], nuclear magnetic resonance (NMR) [11]. It also can offer the possibility of evaluating the quality of TCMs following the overall principle [12,13]. Among these techniques, HPLC has been the most widely used for fingerprint analysis. However, long analysis times were usually required in TCMs analysis in order to obtain a holistic chemical profile for HPLC fingerprints.

Supercritical fluid chromatography (SFC) using supercritical carbon dioxide as the major mobile phase has been applied in TCM analysis as a green technique. SFC displays several advantageous properties such as fast separation and high efficiency, as well as low consumption of organic solvents [14,15]. These features are highly beneficial from a chromatographic point of view to obtain a fast analysis and a good resolution, which can not only avoid the use of great amounts of organic solvents that are both toxic and expensive, but also provide more useful chemical information for SFC fingerprint analysis. However, SFC fingerprints of TCMs often contain highly complex multivariate data that make their interpretation difficult. Therefore, chemical pattern recognition (CPR) methods, such as hierarchical cluster analysis (HCA), principal component analysis (PCA) and partial least squares discriminant analysis (PLS-DA) could be considered as reasonable methods to simplify the complex data [8]. To our knowledge, there are no reports yet on the quality evaluation of *M. bealei* using SFC fingerprints with CPR. 

**Figure 1:**
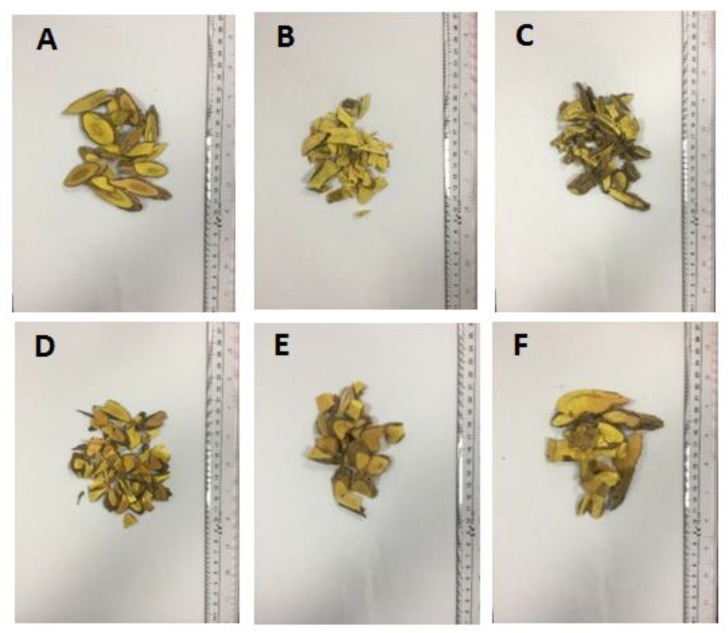
Appearance characters of *M. bealei* samples and its substitutes. **A**: cultivated *M. bealei*; **B**: wild *M. bealei*; **C**: *M. breviracema* Y. S. Wang et Hsiao; **D**: *M. duclouxiana* Gagnep; **E**: *M. bodinieri* Gagnep; F: *M. fordii* Schneid.

The main aim of this study was to establish a reliable method using SFC fingerprints and CPR methods for the quality evaluation of *M. bealei*. For this purpose, wild *M. bealei*, cultivated *M. bealei* and four other *M. bealei* substitutes were chosen as study objects (Figure 1). Unsupervised (HCA and PCA) and supervised (PLS-DA) pattern recognition methods were both applied to analyze the obtained data in order to establish an accurate classification model. Thereafter, the established model was further validated by testing set samples. The predictive ability of the model was also evaluated by the statistical parameters of a prediction set. Interestingly, the method could not just provide an effective and comprehensive way to evaluate the quality of *M. bealei*, but could also allow their differentiation at the chemistry level.

## 2. Results and Discussion

### 2.1. Optimization of the Chromatographic Conditions

Four stationary phases, namely NH_2_, XAmide, Pyridine, and PFP were compared in order to obtain adequate selectivity using appropriate gradient elution (Table S1). As can be seen in Figure S1, the best selectivity was observed for the NH_2_ column. Thereafter, the detection wavelength was also optimized to obtain as much chemical information as possible. In terms of the number of peaks and peak intensity, 230 nm was selected as the optimal wavelength (Figure S2).

*M. bealei* was reported as rich being in alkaloids [16]. Considering the properties of alkaloids, different concentrations of diethylamine (0–0.8%, *v*/*v*) were added to improve the peak shape and selectivity. Finally, the best separation performance, with a resolution (Rs) of 1.64 for the critical pair (peaks 6 and 7, Figure S3) was obtained using 0.4% (*v*/*v*) diethylamine. In order to improve the retention of analytes, the use of a second additive (water) was investigated. It was found that retention was decreased when the amount of this additive was increased from 0 to 8% (*v*/*v*) water, the Rs value for peak 6 and 7 was increased from 1.31 to 2.55. However, the baseline drifted when a concentration of 8% (*v*/*v*) water was used (Figure S4), therefore, 5% (*v*/*v*) water was considered as the best compromise for the separation of analytes.

Backpressure (100–160 bar) and temperature (20–40 °C) were also investigated in order to further enhance the separation performance. It was found that the best Rs of the peaks 6 and 7 were obtained when the temperature and backpressure were set at 28 °C and 140 bar (Figures S5 and S6), respectively. Finally, these optimal conditions were applied to the analysis of extracts of wild *M. bealei*, cultivated *M. bealei* and its substitutes.

### 2.2. Repeatability and Stability Evaluation

The developed SFC fingerprint method was validated for repeatability, and stability. For the evaluation of repeatability six independent samples were prepared in parallel, according to Section 3.3. The relative standard deviations (RSDs) of peak area and retention time (RT) of 11 common peaks were less than 2.3% and 0.24%. The stability was assessed by repeatedly analyzing the same solution after being placed at room temperature for 0, 2, 4, 8, 12, and 24 h. The RSDs of peak area and RT of 11 common peaks were below 2.56% and 0.25%, respectively. These results indicated that the established SFC fingerprint method of *M. bealei* was stable and reliable.

### 2.3. SFC Fingerprint Analysis and Similarity Evaluation

**Figure 2:**
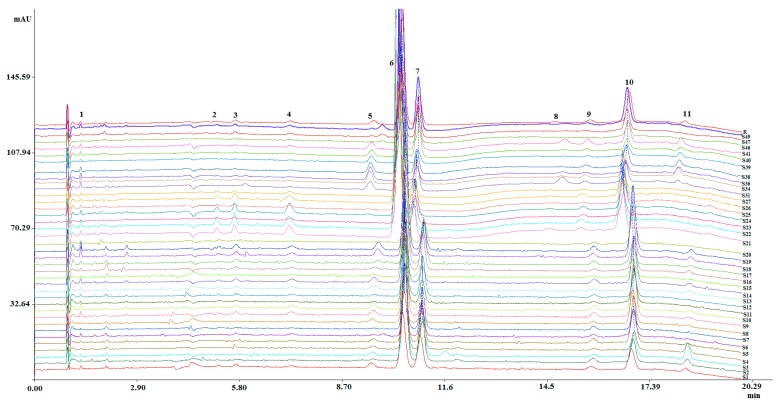
SFC fingerprints of different *M. bealei* samples and its substitutes. Experimental condition: NH_2_ (4.6 mm × 250 mm, 5 μm); mobile phase: (A) sCO_2_; (B) MeOH containing 0.4% (*v*/*v*) diethylamine and 8% (*v*/*v*) water; gradient: 015 min/18%–25% B, 15–20 min/25%–35% B. 20.1–25 min/18% B; injection volume: 5 μL; flow rate: 3.0 mL/min; column temperature: 28 °C; backpressure: 140 bar; detection wavelength: 230 nm; samples: S1–S27, S31, S34, S36, S39–S41, S46–S47, S49.

Thirty seven SFC fingerprints of *M. bealei* (wild and cultivated) and its substitutes from the training set were established under the optimized conditions and then imported into the similarity evaluation system. Sample fingerprints were compared with fingerprint R to generate the similarity values. As a result, 11 common peaks were observed in SFC fingerprints (Figure 2). The similarity values between the generated fingerprint R and analyzed samples were in the range of 0.830 to 0.982 (Table 1). These results indicated that no significant difference was observed between these various samples. However, the contents of 11 common peaks were quite different, that might result in quality differences. Furthermore, CPR methods were employed to assess the variation in quality.

### 2.4. CPR Analysis

#### 2.4.1. HCA and PCA Analysis

**Figure 3:**
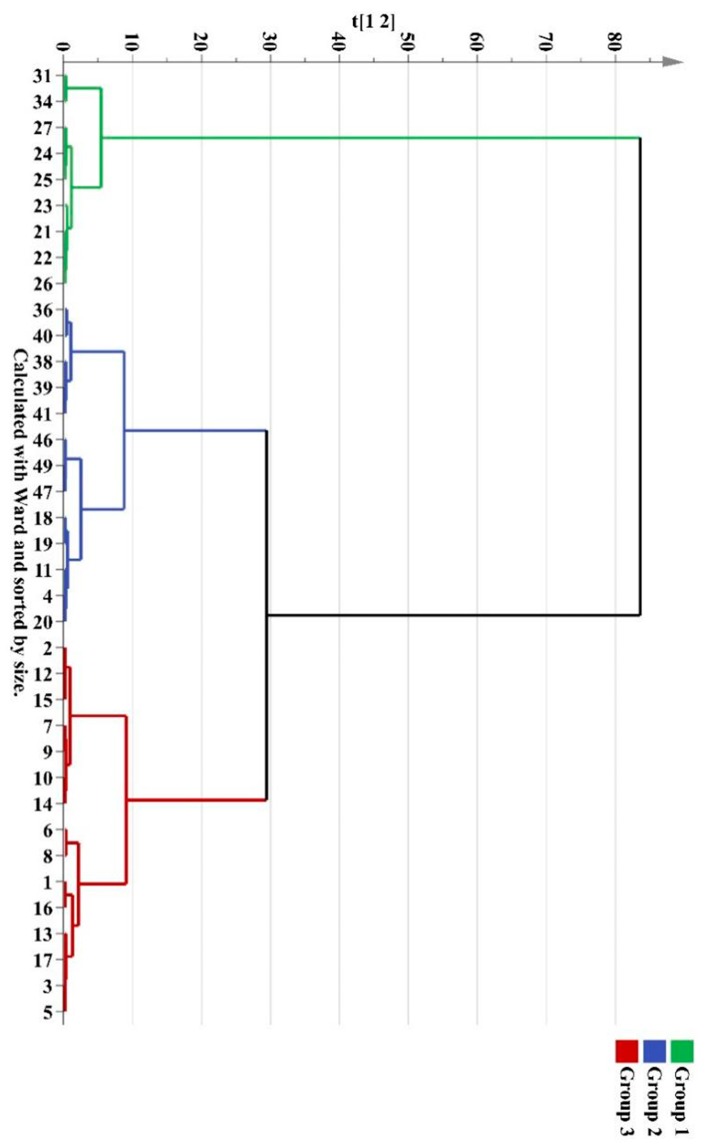
HCA dendrogram of different *M. bealei* samples and its substitutes.

**Figure 4:**
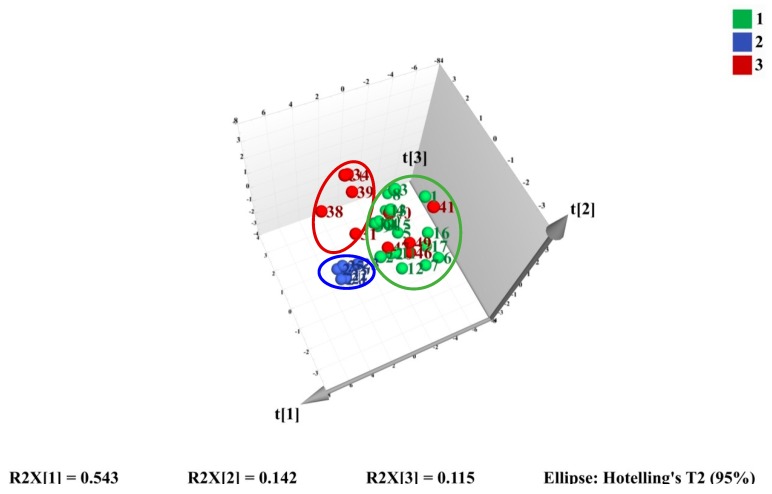
3D score plot of PCA on the first three PCs for training set samples.

To evaluate the difference of the components in wild *M. bealei*, cultivated *M. bealei* and its three types of substitutes, unsupervised HCA and PCA were performed. Thirty seven samples were selected as observations while the peak areas of 11 compounds were selected as variables forming a 37 × 11 data matrix. The corresponding HCA dendrogram and PCA score plot are shown in Figure 3 and Figure 4, where it can be seen that these samples were sorted into three clusters reflecting the quality differences of the three types of samples. Among them, samples 31 and 34 were misclassified into the wild *M. bealei* group, whereas samples 4, 11, 18, 19, and 20 were misclassified into the substitutes. This demonstrated that the 11 characteristic peaks were unable to accurately classify the three clusters.

Subsequently, PCA was employed to investigate if the three types of sample could be differentiated. The 3D score PCA plot is shown in Figure 4. 

The first three principal components (PCs) were extracted and explained 54.3%, 14.2%, and 11.5% of the total variability, respectively. All three PCs could accumulate 80.0% of the data variance. However, appropriate visualization and differentiation could not be observed especially for cultivated *M. bealei* and the substitutes. The results of HCA and PCA indicated that the 11 characteristic peaks were not representative and not specific for satisfactory classification. Consequently, a supervised method is needed to find out the specific variation to classify the three clusters accurately.

#### 2.4.2. PLS-DA Analysis

**Figure 5:**
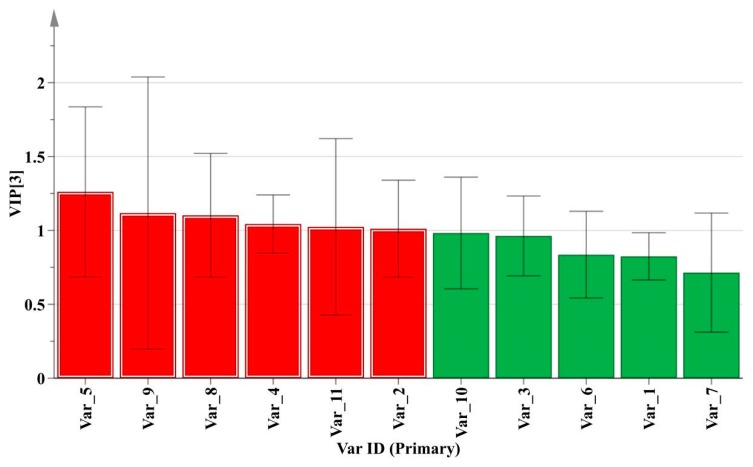
VIP plot for training set samples based on PLS-DA method.

As a supervised recognition pattern, PLS-DA can maximize the difference among the groups and screen out key markers responsible for sample variation [7]. The profile of variable importance in projection (VIP) could usually reflect the contribution levels of some key markers for categories differentiation. The variables with VIP values greater than 1 were considered to be more relevant for sample classification [17]. As shown in Figure 5, the VIP plot was constructed to measure the important variables on sample discrimination. After T-tests for these important variables using SPSS 16.0 software, six significantly correlated chemical markers (red histogram) including peak 5, peak 9, peak 8, peak 4, peak 11, and peak 2 were screened out for classification.

**Figure 6:**
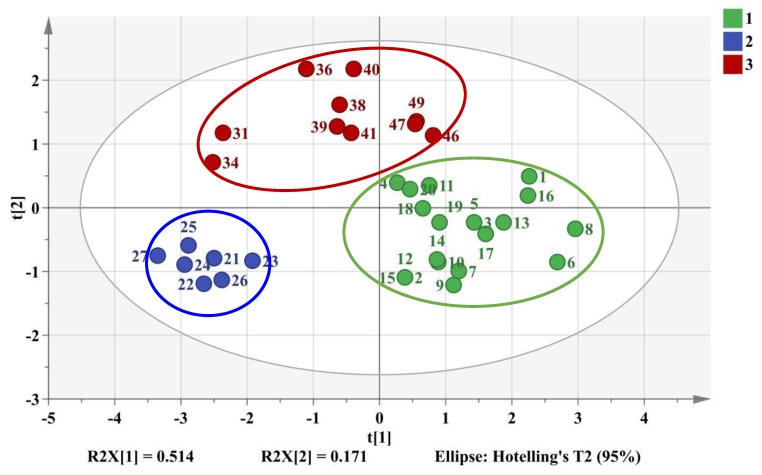
2D PLS-DA score plot of training set samples. Cluster 1 (green color): cultivated *M. bealei*; Cluster 2 (Blue color): wild *M. bealei*; Cluster 3 (red color): substitutes.

Among them, peaks 5, 9, 11 were successfully identified by reference standards as tetrandrine, columbamine, and berbamine with comparison of the RT (Figure S7) and DAD spectrum (data not shown) while the others were unknown compounds from *M. bealei*. The selected optimal chemical markers were further evaluated by a PLS-DA model. A new data of 37 × 6 matrix was formed to reconstruct the classification model. The 2D PLS-DA score plot was shown in Figure 6, the values of R^2^X, R^2^Y, and Q^2^ were 0.685, 0.776, and 0.746 at a confidence level of 95%, respectively. The results indicated that the established model showed a good fitting and predictive ability. The training set samples were accurately sorted into three related clusters: cluster 1 (cultivated *M. bealei*), cluster 2 (wild *M. bealei*) and cluster 3 (substitutes), demonstrating a remarkable difference among the three types. To validate the accuracy of the PLS-DA model, 200 permutation tests were carried out and the vertical intercept values of R^2^ and Q^2^ were 0.0103 and −0.271 (Figure S8), respectively, indicating that the established model avoided an over-fitting problem and showed a good prediction [18].

**Figure 7:**
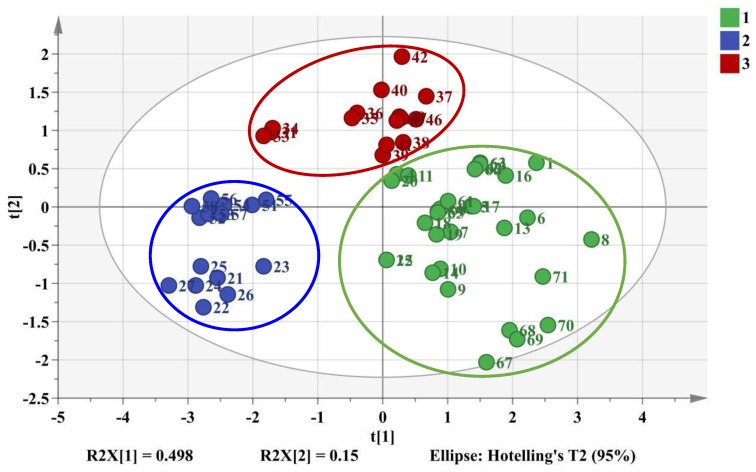
2D PLS-DA score plot of training set samples and testing set samples. Cluster 1 (green color): cultivated *M. bealei*; Cluster 2 (Blue color): wild *M. bealei*; Cluster 3 (red color): substitutes.

Moreover, 26 batches of samples chosen as testing set containing all these three categories were validated. As shown in Figure 7, the values of R^2^X, R^2^Y, and Q^2^ were 0.648, 0.692, and 0.655 at a confidence level of 95%. The testing set samples were also accurately separated into the three related clusters that further verified the success of PLS-DA model. Eventually, PLS-DA model could successfully provide a distinct classification of samples on the basis of the six characteristic peaks compared to HCA and PCA.

In order to prove the predictive ability of PLS-DA model, 26 samples containing 18 cultivated *M. bealei*, two wild *M. bealei* and six substitutes were selected as investigation. As shown in Table 2, the results of the entire data set indicated that the three types of samples were successfully classified with a Fisher’s probability test of 5.6 × 10^−6^. It showed that the prediction results were accurate and the established PLS-DA model could be effectively applied for quality evaluation of *M. bealei* as well as its quality prediction.

## 3. Materials and Methods

### 3.1. Chemicals and Materials

HPLC-grade methanol (MeOH) was purchased from Merck (Darmstadt, Germany). Diethylamine and hydrochloric acid (HCl) were obtained from Guangzhou Chemical Reagent Factory (Guangzhou, China). Three reference standards (tetrandrine, columbamine, berbamine) and reference extract of *M. bealei* were supplied by National Institutes for Food and Drug Control (Beijing, China). Purified water was from a Milli-Q water purification device (Millipore, Billerica, MA, USA). The detailed sample information is listed in Table 3.

All cultivated *M. bealei* samples were authenticated by TCM testing department (Shenzhen Institute for Drug Control, Guangdong, China) and wild *M. bealei* were authenticated by Prof. Weixian Deng (Shaoguan University, Guangdong, China). All the samples were randomly divided into training set, validation set and predication set. As a result, the training set consisted of 37 samples including S1–S27, S31, S34, S36, S39–S42, S46–S47 and S49 to establish a classification model. 26 samples including S33, S35, S37–S38, S48, S51–S71 were used as testing set for model validation while another 26 samples including S28–S30, S32, S43–S45, S50, S72–S89 were applied as prediction set to verify the prediction ability.

### 3.2. Instrumentation

SFC experiments were performed on an 1260 Infinity Hybrid SFC/UHPLC Analytical System consisting of a SFC binary pump, a modified 1260 Infinity LC system, a degasser, a SFC autosampler with 5 μL loop, a SFC Fusion^TM^ A5 module, a thermostatted column compartment and a diode array detector (DAD) with a high pressure SFC flow cell (Agilent Technologies, Santa Clara, CA, USA). Instrument and data collection were performed on Agilent OpenLab ChemStation Edition C.01.05 software. The following columns were applied in this research: Unitary XAmide (150 mm × 4.6 mm, 5 μm) was obtained from Acchrom Technologies (Beijing, China); Sepax SFC-Pyridine column (250 mm × 4.6 mm, 5 μm) was purchased from Sepax Technologies Inc (Delaware, DE, USA); Platisil NH_2_ column (250 mm × 4.6 mm, 5 μm) and Inspire PFP column (250 mm × 4.6 mm, 5 μm) were from Dikma Technologies Inc. (Guangzhou, China). A pulverizer (Fenghang, Shanghai, China) and an ultrasonic water bath (Kun Shan, Jiangsu, China) were used for the extraction process.

### 3.3. Preparation of Sample Solutions

The sample preparation method was carried out following the Chinese Pharmacopoeia protocol (2015 edition) [1]. Dry raw material of *M. bealei* was firstly grounded into powder and then filtered by 30 mesh sieves. All *M. bealei* (0.25 g per each) were then transferred to a 150 mL conical flask with stopper, and 50 mL MeOH/HCl (100/1, *v*/*v*) was added. After ultrasonication at room temperature for 45 min, MeOH/HCl (100/1, *v*/*v*) was added to compensate the weight loss during extraction. All the solutions were filtered through a 0.22 μm membrane and then stored at 4 °C prior to analysis.

### 3.4. Chromatographic Conditions

The final SFC experiment was performed on Platisil NH_2_ (4.6 mm × 250 mm, 5 μm) at a flow rate of 3 mL/min. The mobile phase consisted of supercritical carbon dioxide (sCO_2_, component A) and MeOH containing 0.4% (*v*/*v*) diethylamine and 8% (*v*/*v*) water (component B). The gradient elution was as follows: 0–15 min/18%–25% B, 15–20 min/25%–35% B. 20.1–25 min/18% B. The column temperature and backpressure were set at 28 °C and 140 bar, respectively. The injection volume was 5 μL while the detection wavelength was set at 230 nm.

### 3.5. Data Analysis

#### 3.5.1. Similarity Analysis (SA)

The raw SFC chromatographic data of the 37 tested samples were integrated automatically and exported as *.AIA format file. SA was performed using the software “Similarity Evaluation System for Chromatographic Fingerprint of Traditional Chinese Medicine” (Version 2004 A, Chinese Pharmacopoeia Committee) with these exported files. The reference extract of *M. bealei* as control (fingerprint R) was generated and the similarity values of these samples were then calculated.

#### 3.5.2. CPR Analysis

Before performing CPR analysis, the data were normalized using Z-score transformation method (SPSS 16.0 software, IBM Inc., Chicago, IL, USA). Afterwards, CPR analysis was performed using HCA, PCA, and PLS-DA, which were carried out by SIMCA-P 14.0 software (Umetrics AB, Umea, Sweden). In HCA, a dendrogram was obtained to characterize the classification result of the samples by Ward’s linkage as cluster method. The sample variation could be assessed by PCA while PLS-DA was used to screen out the main markers that were responsible for discrimination. The parameters of the modeling (R^2^ and Q^2^ values) explain the quality of the fitting model. R^2^ estimates the fitness of the model while Q^2^ estimates the ability of prediction. Large R^2^ and Q^2^ (>0.5) indicate a model with satisfying fitness and predictivity [19]. When PLS-DA is used as a CPR method, the samples are usually divided into a training set, testing set and prediction set [20]. The classification model is obtained by training set and the model is validated by testing set and prediction set.

## 4. Conclusions

A comprehensive method was developed combining SFC fingerprints and CPR to evaluate the quality of *M. bealei*. The fingerprints were established and 11 common peaks were obtained among 37 samples. Three types of sample (wild, cultivated and substitutes) presented quite similar chemical composition through SA study which made their classification difficult. Therefore, CPR methods (HCA, PCA and PLS-DA) were applied to evaluate the quality based on the 11 chemical compounds. Results revealed that HCA and PCA were not able to provide an accurate classification for the three selected types of sample which indicated that the 11 compounds were not representative and specific for accurate classification. PLS-DA was then applied for feature extraction from 11 compounds in order to find some important variables that are responsible for the accurate classification. Six characteristic peaks (5, 9, 8, 4, 11, 2) with VIP values greater than 1 were screened. Among them, peaks 5, 9 and 11 were successfully identified as tetrandrine, columbamine, and berbamine. Moreover, the three types of sample were accurately divided into three categories representing wild *M. bealei*, cultivated *M. bealei* and its substitutes. The established model was then successfully validated by testing set samples and predication set samples. The results indicated that the accurate discrimination of the three types of samples could be obtained using PLS-DA model. In conclusion, the developed SFC fingerprint method coupled with PLS-DA can be considered as a promising approach for the quality evaluation of *M. bealei*.

## Figures and Tables

**Table 1:** SA results of 37 batches of *M. bealei* samples and its substitutes.

Sample	Similarity	Sample	Similarity
**S1**	0.942	**S20**	0.936
**S2**	0.942	**S21**	0.870
**S3**	0.955	**S22**	0.871
**S4**	0.952	**S23**	0.898
**S5**	0.957	**S24**	0.861
**S6**	0.964	**S25**	0.862
**S7**	0.976	**S26**	0.875
**S8**	0.862	**S27**	0.850
**S9**	0.909	**S31**	0.912
**S10**	0.910	**S34**	0.913
**S11**	0.941	**S36**	0.841
**S12**	0.968	**S38**	0.912
**S13**	0.830	**S39**	0.896
**S14**	0.913	**S40**	0.952
**S15**	0.956	**S41**	0.980
**S16**	0.973	**S46**	0.954
**S17**	0.982	**S47**	0.973
**S18**	0.928	**S49**	0.955
**S19**	0.929		

**Table 2:** Prediction results for 26 batches of samples.

	Members	Correct	Cultivated *M. bealei*	Wild *M. bealei*	Substitutes
**Cultivated** ***M. bealei***	18	100%	18	0	0
**Wild** ***M. bealei***	2	100%	0	2	0
**Substitutes**	6	100%	0	0	6
**No class**	0	/	0	0	0
**Total**	26	100%	18	2	6
**Fisher’s probability**	5.6 × 10^−6^				

**Table 3:** Detailed information of samples.

Sample No.	Species	Origin	Specification
1–12	*M. bealei* (Fort.) Carr.	Guangxi	Crude drugs (cultivated)
13	*M. bealei* (Fort.) Carr.	Yunnan	Crude drugs (cultivated)
14	*M. bealei* (Fort.) Carr.	Anhui	Crude drugs (cultivated)
15	*M. bealei* (Fort.) Carr.	Zhejiang	Crude drugs (cultivated)
16	*M. bealei* (Fort.) Carr.	Jiangxi	Crude drugs (cultivated)
17	*M. bealei* (Fort.) Carr.	Sichuan	Crude drugs (cultivated)
18–20	*M. bealei* (Fort.) Carr.	Guizhou	Crude drugs (cultivated)
21–29	*M. bealei* (Fort.) Carr.	Guangxi	Crude drugs (wild)
30–34	*M. breviracema* Y. S. Wang et Hsiao	Guangxi	Crude drugs (wild)
35–39	*M. duclouxiana* Gagnep	Guangxi	Crude drugs (wild)
40–45	*M. bodinieri* Gagnep	Guangxi	Crude drugs (wild)
46–50	*M. fordii* Schneid	Guangxi	Crude drugs (wild)
51–58	*M. bealei* (Fort.) Carr.	Guangdong	Crude drugs (wild)
59–65	*M. bealei* (Fort.) Carr.	Anhui	Crude drugs (cultivated)
66–72, 74–75, 79	*M. bealei* (Fort.) Carr.	Jiangxi	Crude drugs (cultivated)
73	*M. bealei* (Fort.) Carr.	Anhui	Crude drugs (cultivated)
76–78, 80–83	*M. bealei* (Fort.) Carr.	Guangdong	Crude drugs (cultivated)
84	*M. bealei* (Fort.) Carr.	Jiangxi	Crude drugs (cultivated)
85–86	*M. bealei* (Fort.) Carr.	Anhui	Crude drugs (cultivated)
87–89	*M. bealei* (Fort.) Carr.	Guangxi	Crude drugs (cultivated)

## Supplementary Materials

The supplementary materials are available online.
