# Supplementary material for: Quality Evaluation of Mahonia bealei (Fort.) Carr. Using Supercritical Fluid Chromatography with Chemical Pattern Recognition
Source: Molecules. 2019 Oct 13;24(20):3684. doi: 10.3390/molecules24203684 (PMC6832872; doi:10.3390/molecules24203684)
Supplement: Supplementary file 1 [file molecules-24-03684-s001.zip › molecules-597923-SI.pdf]

Supplementary materials

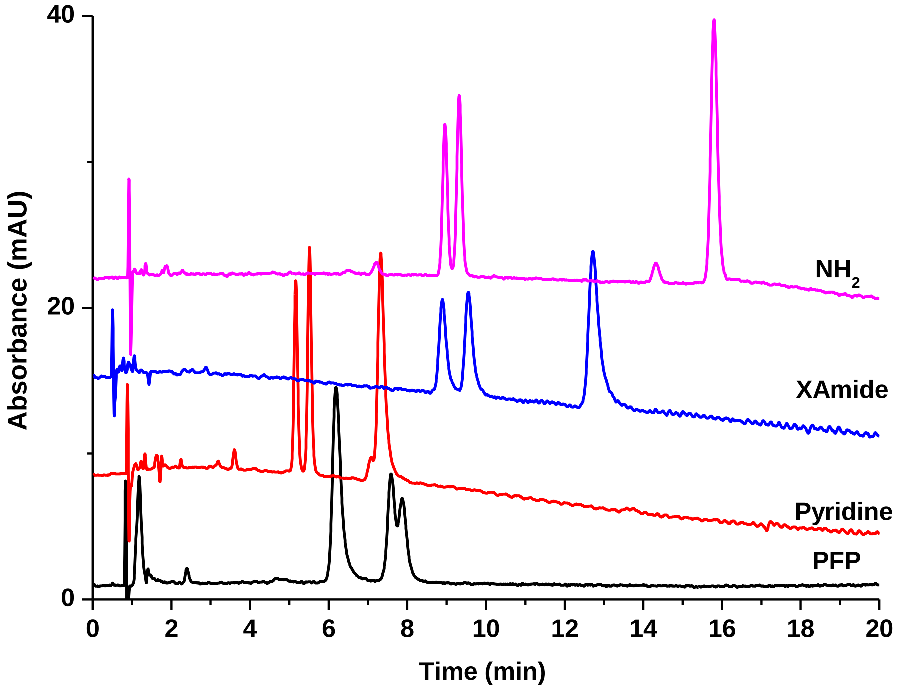

Fig. S1. SFC chromatograms of the extract of *M. bealei* on four different columns. Experimental condition: mobile phase: (A) sCO_2; (B) MeOH containing 0.4% (v/v) diethylamine and 5% (v/v) water; gradient: NH_2,, 0-15 min; 18-25% B; 15-20 min, 25-35% B; XAmide, 0-20 min; 24%-40% B; Pyridine, 0-8 min; 24%-30% B; 8-20 min; 30-40% B; PFP, isocratic elution at 20% B; injection volume: 5 µL; flow rate: 3.0 mL/min; column temperature: 28 °C; backpressure: 140 bar; detection wavelength: 230 nm.

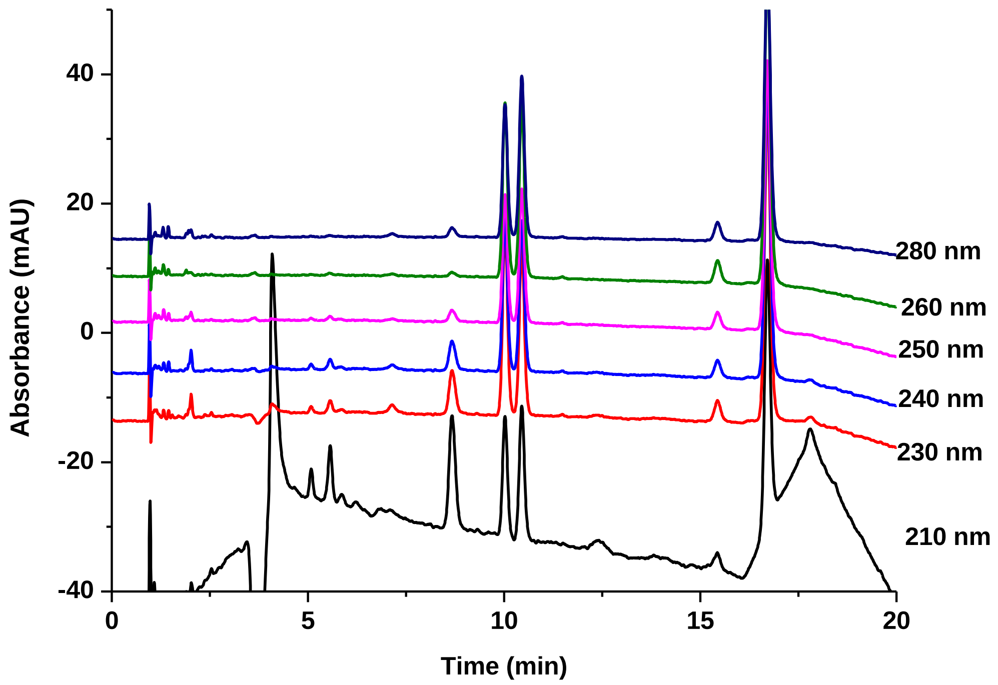

Fig. S2. SFC chromatograms of the extract of *M. bealei* in different wavelength. Experimental conditions: column: NH_2 (4.6 mm × 250 mm, 5 μm); detection wavelength: 210 nm-280 nm; other conditions as in **Fig. S1.**

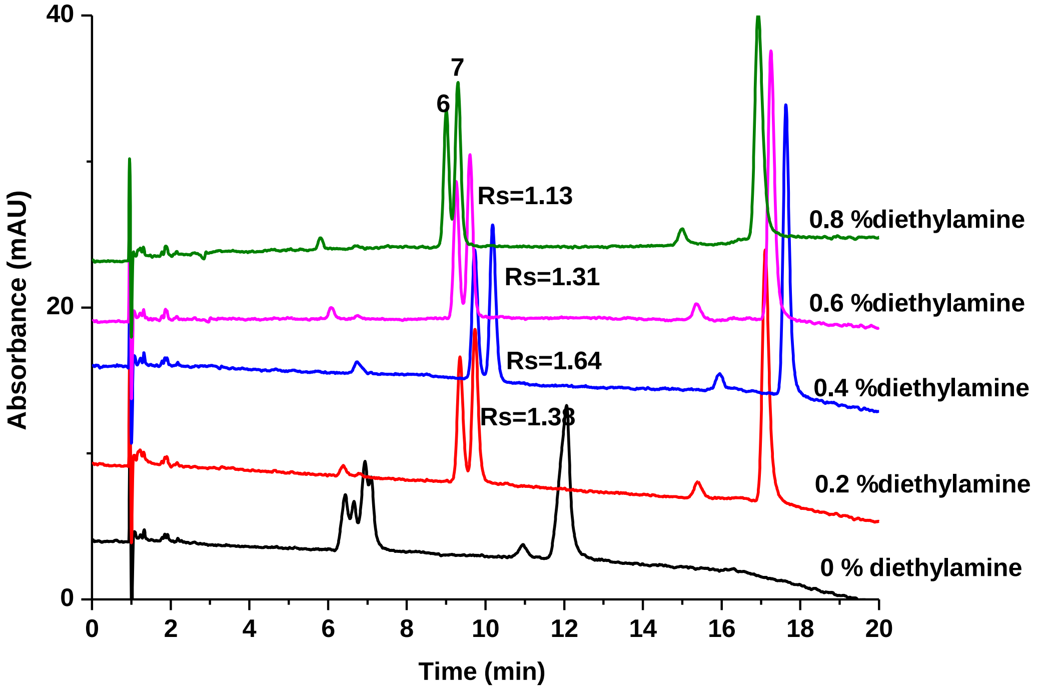

Fig. S3. Effect of the diethylamine concentration in mobile phase on the separation of extract of *M. bealei*. Experimental conditions: column: NH_2 (4.6 mm × 250 mm, 5 μm); other conditions as in **Fig. S1.**

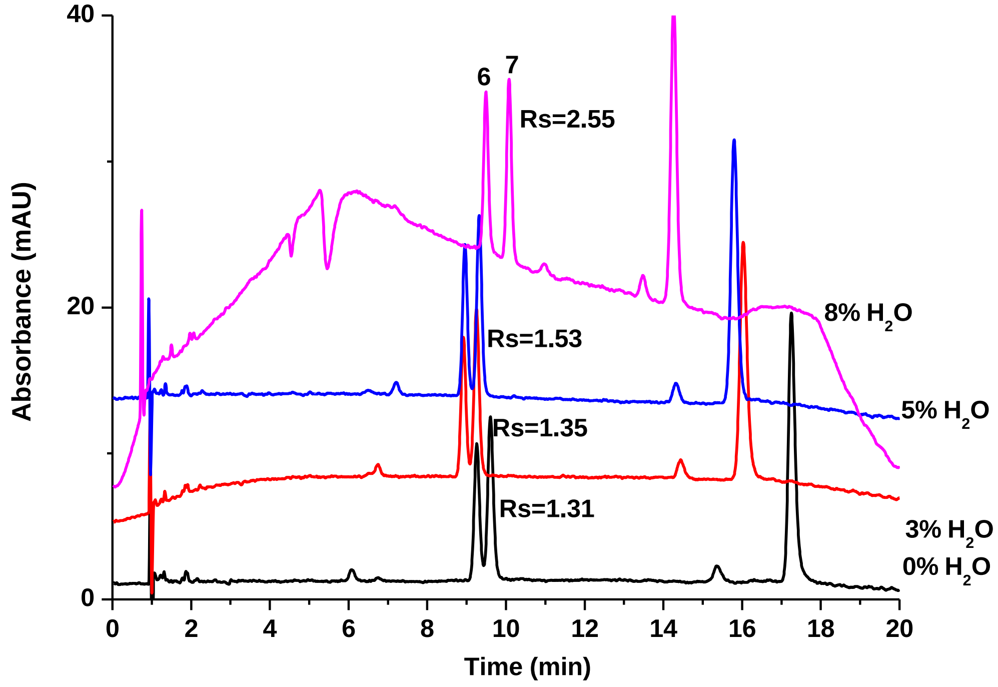

Fig. S4. Effect of the water content in mobile phase on the separation of extract of *M. bealei*. Experimental conditions: column: NH_2 (4.6 mm × 250 mm, 5 μm); other conditions as in **Fig. S1.**

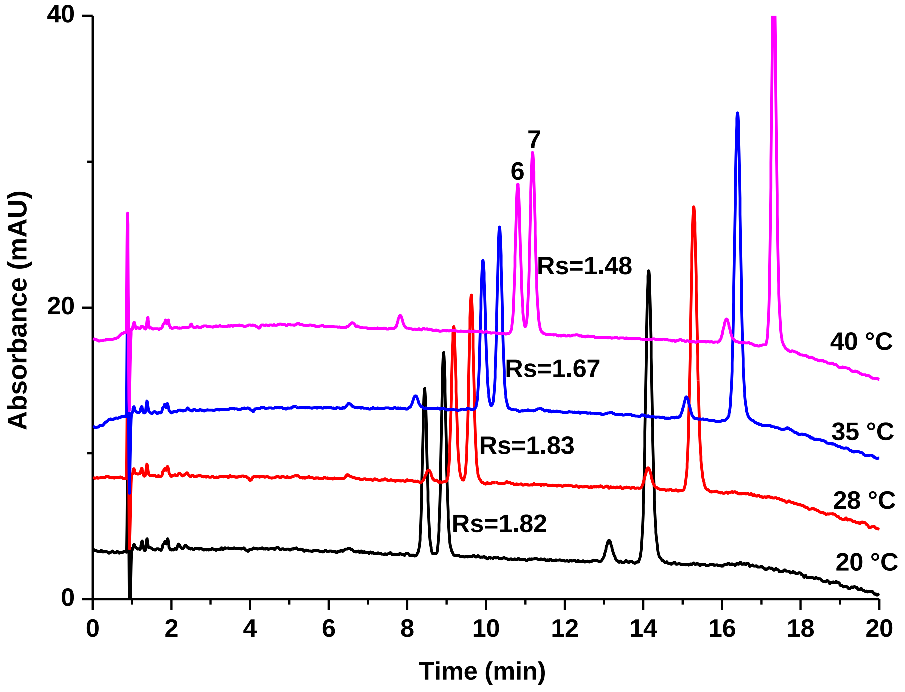

Fig. S5. Effect of temperature on resolution. Experimental conditions: column: NH_2 (4.6 mm × 250 mm, 5 μm); backpressure: 140 bar; other conditions as in **Fig. S1.**

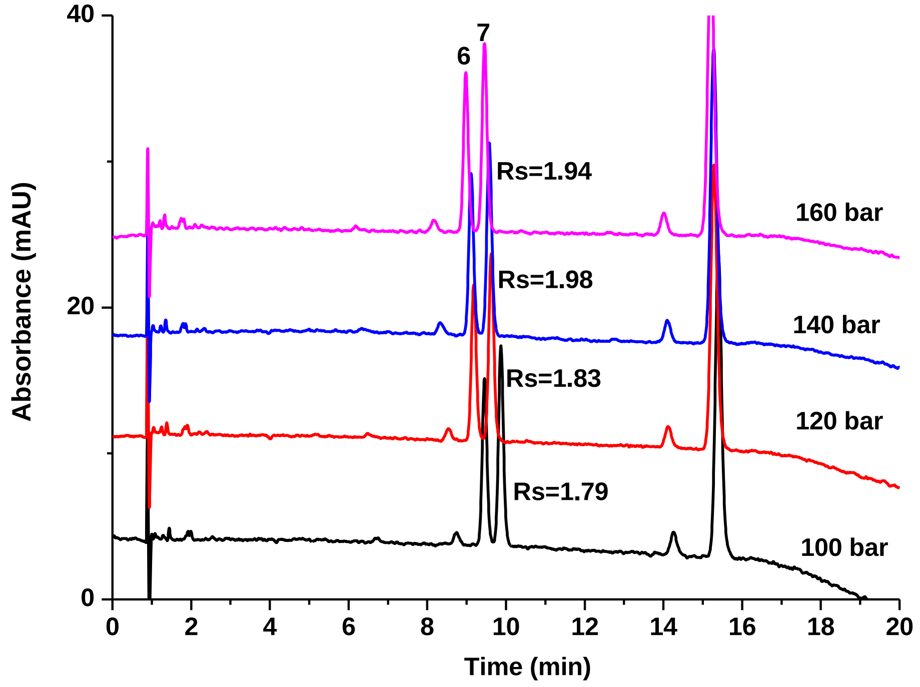

Fig. S6. Effect of backpressure on resolution. Experimental conditions: column: NH_2 (4.6 mm × 250 mm, 5 µm); temperature: 28 °C; other conditions as in **Fig. S1.**

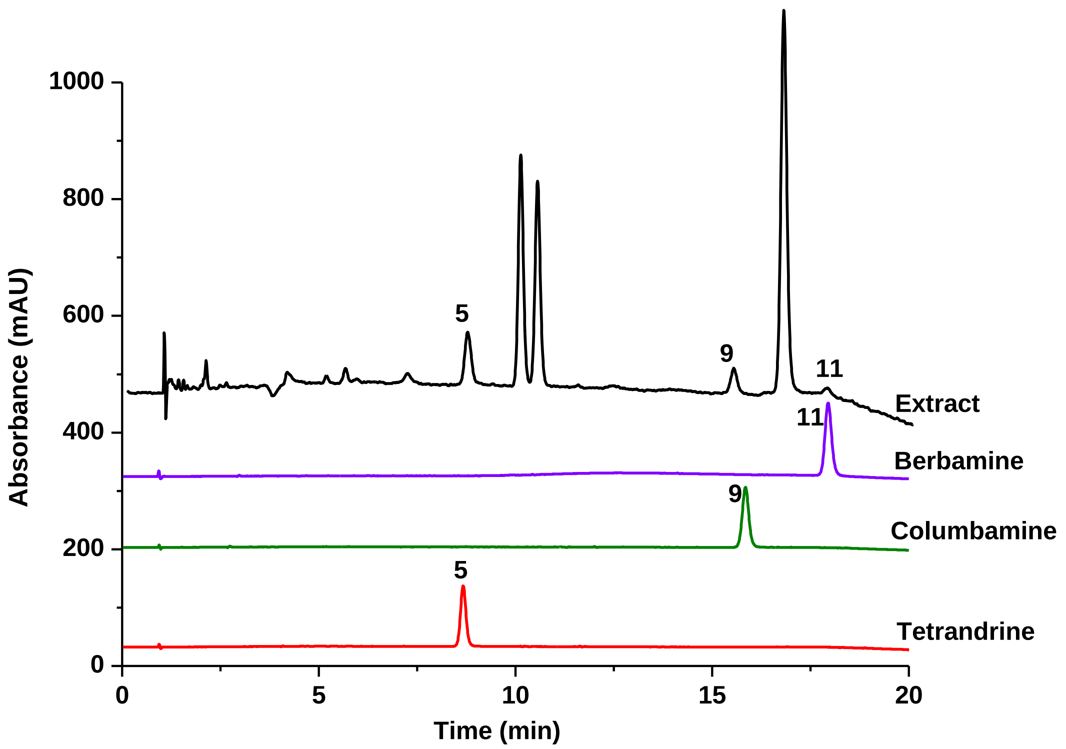

Fig. S7. Identification of three characteristic peaks by reference standards. Experimental conditions: column: NH_2 (4.6 mm × 250 mm, 5 μm); compounds: 5. Tetrandrine; 9. Columbamine; 11. Berbamine; other conditions as in **Fig. S1**.

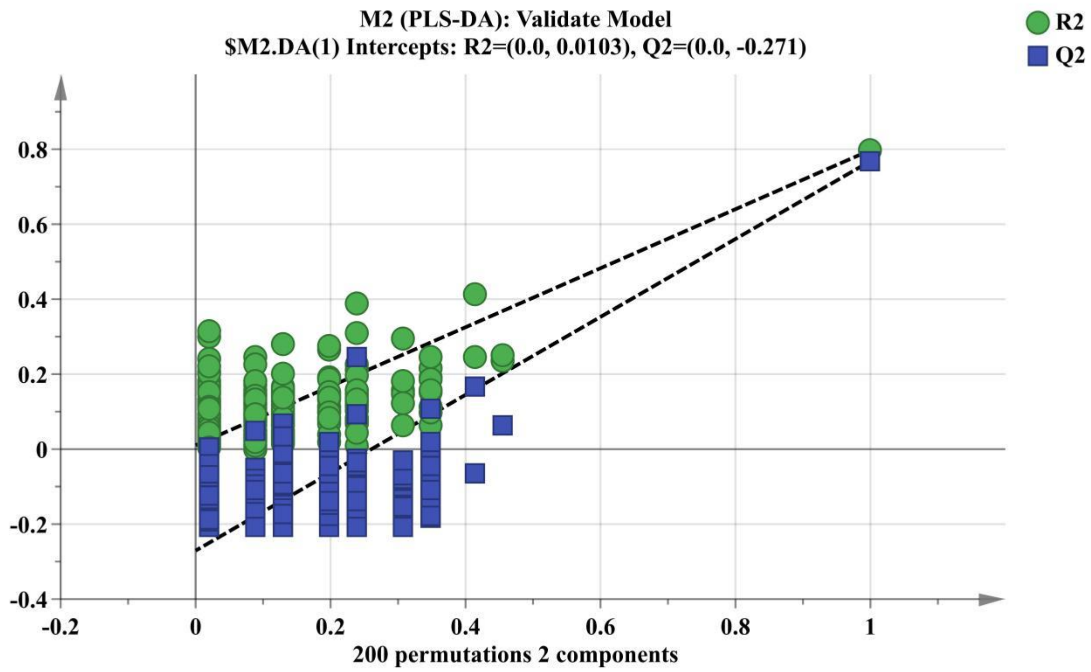

Fig. S8. 200 permutations for the validation of PLS-DA model.
